# What Rang the Telephone Bells?

**Published:** 1891-02

**Authors:** 


### WHAT RANG THE TELEPHONE BELLS?

Last fall, A. M. Taylor, of Summitville, Ind., put up one of his mechanical telephone lines connecting two houses on my farm one hundred rods apart. One of the houses is occupied by the family of A. G. Hill and the other by John Lemasters. The diaphragm of this telephone is enclosed in a frame of wood six or eight inches square, which frame is attached to the side of the room by a stiff spring shaped like letter V, the lower end of the frame being screwed to one of the upper ends of the spring, and the other end of the spring screwed to the wall. It will be seen that a blow upon the upper part of the frame will force the two ends of the spring nearer together, and the recoil of the spring causes a sufficient vibration of the wire attached to the diaphragm to ring the bells which are attached to either frame by a short stiff wire.

A musical instrument played in either house, can be plainly heard at the other. The call is made by a slight blow of the hand upon the upper part of the frame—the more forcible the blow, the louder and longer the bells ring. Last month, one night between 12 and 1, both families were quickly aroused from sound sleep by a violent ringing of the bells, which continued to ring until Lemasters got to the telephone. He asked Hill what he was ringing for. Hill, who had also gone to the instrument at his end of the line replied that he had not rung, and after a remark or two passed expressive of suprise, as there was no wind stirring, both returned to bed. No sooner had Lemasters lain down, than he heard a crackling sound, which he attributed to a horse in a lot near by rubbing against the fence; but the continuance of the noise caused him to get up and go out and in the direction of the animal, when he discovered his house was on fire. Two of the rafters had already burned in two and the flames had reached a distance of six or eight feet from the flue where it began. The moon and stars were shining brightly so he had not noticed the light of the fire or any reflection of it,until he got out into the yard. At one time before this, the bell of the telephone rang lightly, and the cause was discovered to have been the flying of a bird against the wire, the result being the death of the bird. If a large bird had become entangled in the wire, its fluttering might have caused the ringing, but this theory is not a likely one. The statement of the occurrence is perfectly reliable—neither of the men has any explanation to suggest.

J. N. G., in the *R. P. Journal.*

Virginia, Ill.

